# Metformin: A Potential Therapeutic Agent for Recurrent Colon Cancer

**DOI:** 10.1371/journal.pone.0084369

**Published:** 2014-01-20

**Authors:** Pratima Nangia-Makker, Yingjie Yu, Anita Vasudevan, Lulu Farhana, Sindhu G. Rajendra, Edi Levi, Adhip P. N. Majumdar

**Affiliations:** 1 Veterans Affairs Medical Center, Wayne State University, Detroit, Michigan, United States of America; 2 Karmanos Cancer Institute, Wayne State University, Detroit, Michigan, United States of America; 3 Department of Internal Medicine, Wayne State University, Detroit, Michigan, United States of America; University of Kansas School of Medicine, United States of America

## Abstract

Accumulating evidence suggests that metformin, a biguanide class of anti-diabetic drugs, possesses anti-cancer properties. However, most of the studies to evaluate therapeutic efficacy of metformin have been on primary cancer. No information is available whether metformin could be effectively used for recurrent cancer, specifically colorectal cancer (CRC) that affects up to 50% of patients treated by conventional chemotherapies. Although the reasons for recurrence are not fully understood, it is thought to be due to re-emergence of chemotherapy-resistant cancer stem/stem-like cells (CSCs/CSLCs). Therefore, development of non-toxic treatment strategies targeting CSCs would be of significant therapeutic benefit.

In the current investigation, we have examined the effectiveness of metformin, in combination with 5-fluorouracil and oxaliplatin (FuOx), the mainstay of colon cancer therapeutics, on survival of chemo-resistant colon cancer cells that are highly enriched in CSCs/CSLCs. Our data show that metformin acts synergistically with FuOx to (a) induce cell death in chemo resistant (CR) HT-29 and HCT-116 colon cancer cells, (b) inhibit colonospheres formation and (c) enhance colonospheres disintegration. *In vitro* cell culture studies have further demonstrated that the combinatorial treatment inhibits migration of CR colon cancer cells. These changes were associated with increased miRNA 145 and reduction in miRNA 21. Wnt/β-catenin signaling pathway was also down-regulated indicating its pivotal role in regulating the growth of CR colon cancer cells. Data from SCID mice xenograft model of CR HCT-116 and CR HT-29 cells show that the combination of metformin and FuOX is highly effective in inhibiting the growth of colon tumors as evidenced by ∼50% inhibition in growth following 5 weeks of combination treatment, when compared with the vehicle treated controls. Our current data suggest that metformin together with conventional chemotherapy could be an effective treatment regimen for recurring colorectal cancer (CRC).

## Introduction

Recent understanding of the heterogeneous makeup of the cancer cells in a tumor has revealed the presence of CSCs/CSLCs [1], [2], which exhibit self-renewing characteristics, ability to initiate tumor from a small number of cells that are highly chemo-resistant [3]–[7]. Carcinoma recurrence is in part due to fact that conventional chemotherapy only targets the rapidly dividing cells that form bulk of the tumor, but spares the CSCs/CSLCs [8]. The proportion of CSCs is reported to increase after conventional chemotherapy [9]. Thus, presence of chemotherapy resistant CSCs/CSLCs in the primary tumor may in part be responsible for a failure of complete eradication of tumor resulting in its recurrence at the primary and secondary sites. Development of novel therapeutic strategies, which specifically target CSCs/CSLCs is, therefore, warranted.

Metformin, (1,1-dimethylbiguanide hydrochloride) a FDA approved biguanide anti-diabetic drug, is derived from French lilac (*Galega officinalis*), a plant used in folk medicine for several centuries. In addition to its function as a gluconeogenesis suppressor, metformin has recently been shown to possess strong anti-cancer properties. In type 2 diabetic patients metformin has been shown to suppress carcinogenesis in breast, pancreas and lung, and to diminish cancer related mortality (reviewed in [10], [11]. In a number of preclinical studies, metformin reduced proliferation, induced apoptosis, caused cell cycle arrest, and reduced incidence and growth of experimental tumors *in vivo* [12]–[18]. Some reports also indicate that metformin improved the response of human breast tumor xenografts to conventional chemotherapy by eradicating CSCs in the tumor [19], [20].

Many investigators have reported the pivotal role of Wnt/β-catenin signaling pathway in the regulation of epithelial stem cell self-renewal [21], [22], and its dysregulation has been implicated in many malignancies including colorectal cancer (CRC) [23], [24]. In CRC, as a result of inactivation of APC gene, the coordinated phosphorylation and destruction of β-catenin is disrupted. As a result, β-catenin accumulates in the cytoplasm, complexes with DNA-binding proteins of TCF/LEF family and translocates to the nucleus [25], [26]. Once there, it activates the transcription of its target genes like cyclin D1, c-myc, MMP-7, MT1-MMP, axin1 etc. [27]. Many of these target genes are implicated in intestinal stem cell proliferation and carcinogenesis [28]. Recently we showed that Wnt/β-catenin pathway plays a critical role in growth and maintenance of colonospheres, which are highly enriched in CSC/CSL cells [29]. Increased levels of β-catenin in colonospheres were associated with induction of transcriptional activation of TCF/LEF, which was decreased when β-catenin was silenced using siRNA [29].

A novel class of short non-coding RNAs, so called miRNAs has emerged as a regulator of mRNA translation. A miRNA can act as a tumor suppressor or an oncogene depending on its targets in different tissues and cell types [30]. Comprehensive analyses of miRNA-expression patterns in human cancers have revealed that different cancer types have distinct miRNA expression patterns [31].We have reported that miR21, which has been shown to be up-regulated in CRC [32], induces stemness in chemo-resistant (CR) colon cancer cells [33]. miR21 has been shown to regulate the growth, migration, invasion and apoptosis related properties of cancer cells [34]. Down-regulation of miR21 by CDF, an analog of curcumin [35], resulted in normalization of PTEN/Akt axis in CR HCT-116 and CR HT-29 cells and reduced growth [36]. Over-expression of miR21in HCT-116 cells resulted in an increased β-catenin activity [33].

The present investigation was undertaken to examine whether metformin could be used in conjunction with conventional chemotherapy to inhibit the growth of chemo-resistant colon cancer cells and the regulation of this process. Herein, we demonstrate that metformin acts synergistically with FuOx, a combination of 5-FU and oxaliplatin, the mainstay in colon cancer chemotherapy to inhibit the growth of colon CR cells *in vitro* and *in vivo* as well as their migration via down-regulating miR21and inhibiting the Wnt/β-catenin signaling pathway.

## Materials and Methods

### Cell lines and Reagents

Human colon cancer cells HT-29 and HCT-116 were obtained from the American Type Culture Collection (ATCC, Rockville, MD). The cells were maintained in Dulbecco's modified Eagle's medium (4.5 g/I D-glucose) supplemented with 10% fetal bovine serum (Invitrogen, Grand Island, NY) and 1% antibiotic/antimycotic in humidified incubator at 37°C in an atmosphere of 95% air and 5% carbon diaoxide. 5-Fluorouracil + Oxaliplatin (FuOx) resistant cells (CR cells) were generated as described earlier [37]–[39] in our laboratory. The CR cells were maintained in normal culture medium containing 2× FuOx (50 µM 5 FU + 1.25 µM Ox). Endothelial cells were a gift from Dr Dipak Banerjee, University of Puerto Rico and maintained in Earle's Minimum Essential medium supplemented with 10% fetal bovine serum (Hyclone) as previously described [40]. The medium was changed two times a week, and cells were passaged using 0.05% trypsin/EDTA (Invitrogen). The use of cell lines was approved by the Human Investigation Committee, Wayne State University, Detroit, MI. Metformin hydrochloride was purchased from Sigma Chemical Co. A 2 M solution was prepared in sterile distilled water and stored at −20°C. FU and Ox were obtained from Sigma Chemical Co.

### Determination of cellular growth

The growth of colon cancer cells was assessed by mitochondrial-dependent reduction of 3-(4,5-dimethylthiazol-2yl)-2, 5-diphenyltetrazolium bromide (MTT) (Sigma) to formazan as described previously [41]. Briefly, the cells (5×10^3^) were seeded in quadruplicates onto 24 well culture dishes. After 24 hr, fresh medium containing various concentrations of metformin and/or FuOx was added. After 72 hr, cell proliferation was determined by MTT assay. Briefly, the medium was removed and the cells were incubated at 37°C with MTT (0.5 mg/mL) for 4 hr. The medium was aspirated and the cells were solubilized in 0.04 N HCl in isopropanol. The optical density (OD) was measured at 570 and 630 nm.

### Analysis of interaction between metformin and FuOx

Combination indices (CI) method adapted for *in vitro* drug testing was employed to determine the nature of interaction between metformin and FuOx. This method utilizes multiple drug effect equation originally derived from enzyme kinetics method, where the output is represented as CI and/or isobologram analysis.CI analysis was performed by utilizing Calcusyn software (Biosoft, Ferguson, MO). Based on CI values, extent of synergism/anatogonism is determined. In general, CI values below 1 suggest synergy, whereas CI values above 1 indicate antagonism between the drugs. CI values in the range of 0.9–1.10 would mainly indicate additive effects: those between 0.9–0.85 suggest slight synergy, and values in the range of 0.7–0.3 are indicative of moderate synergy. Any values less than 0.3 would suggest strong synergistic interactions between the drugs.

### Formation and differentiation of colonospheres

The ability of cells to form spheres in suspension was evaluated as described by Liu et al [42] with slight modifications [29], [43]. Briefly, colonospheres were generated by incubating a limited number of parental and CR HCT-116 and HT-29 cells at a concentration of 100 cells per 200 µL in serum -free stem cell medium (SCM) containing DMEM/F12 (1∶1) supplemented with B27 (Life technologies, Gaithersberg, MD) 20 ng/mL epidermal growth factor (Sigma, St. Louis, MO), 10 ng/mL fibroblast growth factor (Sigma) and antibiotic/antimycotic in 24 well plates in the presence of metformin alone or in combination with FuOx. The colonospheres formed after 8 days were evaluated for their size and number by light microscopy. In one set of experiments, the colonospheres formed in normal medium were treated with metformin and/or FuOx to analyze their effect on sphere differentiation and attachment.

### Extreme limiting dilution analysis

Extreme limiting dilution analysis (ELDA) was performed as described by Hu and Smyth [44]. Briefly, single cell suspension obtained from adherent cells pretreated with metformin and/or FuOx for 72 hr were plated at a concentration of 100, 10 and 1 cell per 100 µLSCM (24 well for each dilution) in 96 well plates and incubated for 8 days. At the end of 8 days, the number of wells showing formation of colonospheres was counted. The frequency of sphere forming cells was determined using ELDA webtool at http://bioinf.wehi.edu.au/software/elda.

### Cell migration assay

This assay was performed using a Boyden chamber (Neuroprobe Inc., Cabin John, MD) as described earlier [41], [45]. In the lower chamber 100 µg/mL Matrigel (BD Biosciences, Bedford, MA) alone or mixed with metformin and/or FUOX was added. HT-29 or CR HT-29 cells (5×10^4^) cells were loaded in the upper chamber. The two chambers were separated by a polycarbonate filter of 8 µ pore size and incubated in a 37°C tissue culture incubator for 16 hr, after which the filter was removed, the cells on top of the filter were wiped off and the migrated cells were fixed, stained using Protocol Hema 3 stain set (Fisher Scientific Company, Pittsburgh, PA). The migrated cells were photographed using Olympus 45 microscope supporting a Spot Idea camera and the migrated cells per field were counted. Each assay was carried out thrice and 6 wells were used for each treatment.

In the next set of experiments, migration of colon cancer cells towards endothelial cells was measured. In this investigation, enodothelial cells or HT-29/CR HT-29 cells (2.4×10^4^) were pre-labeled with viable stain DiO or DiI respectively (Invitrogen), washed twice with complete medium and seeded in each chamber of the cell culture insert (ibidi GmbH). After 24 hr, the cell culture insert was removed and the migration of the co-cultures towards each other for wound healing was observed. In some cultures, metformin and/or FuOx was added at the time of removal of the insert. The cells were photographed at 0, 24 and 48 hr after removal of the insert.

### Western blot analysis

Western blot analysis was performed according to the standard protocol [46]. Briefly, the cells were solubilized in lysis buffer (50 mM Tris HCl, pH 7.4, 150 mM NaCl, 5 mM EDTA, 1% Triton X100, 0.5% NP40, 25 µg/mL each of aprotinin, leupeptin and pepstatin, 1× phosphatase inhibitor cocktail (Sigma). After clarification at 10,000 g for 30 min, the supernatant was used for protein analysis. Protein concentration determined by the Bio-Rad protein assay kit ((Bio-Rad, Hercules, CA) and aliquots containing 25 µg protein were separated by SDS-polyacrylamide gel electrophoresis. After electrophoresis, proteins were transferred to a polyvinylidene difluoride membrane (Millipore) by electroblotting and subjected to western blot analysis with the recommended dilution of primary antibody. Following washes the blots were reacted with secondary antibody mix containing 1∶2500 dilutions of the appropriate horseradish peroxidase-conjugated secondary antibody (Amersham Biosciences). Protein bands were visualized using a commercially available enhanced chemiluminiscence kit (Amersham Biosciences). Blots were also immuno-reacted with a 1∶5000 dilution of anti-actin mouse monoclonal antibody (Santa Cruz Biotechnology Inc., Santa Cruz, CA) to normalize for variation in protein loading.

### Isolation of RNA and Quantitative Polymerase Chain Reaction analysis

Total RNA was extracted from parental and CR cells treated with metformin and/or FuOx for 48 hr using TRIZOL reagent (Invitrogen) according to manufacturer's instructions. Total RNA was treated with DNase1 to remove contaminating genomic DNA, subsequently purified using miRNAeasy Mini Kit (Qiagen, Valencia, CA). RNA concentration was measured at an optical density of 260 nm.

To quantitate miR-21 and miR-145, first the cDNA synthesis was carried out using Taqman MicroRNA Reverse transcription kit (Applied Biosystems, Foster City, CA). The miRNA RT-PCR primers for miR21, miR145 and endogenous control RNU6B were purchased from Applied Biosystems. Real time qRT-PCR analysis was carried out using Applied Biosystems 7500 real time PCR System. The PCR mix containing Taqman 2× Universal PCR Master Mix were processed as follows: 95°C for 10 min followed by 40 cycles of 95°C for 15 sec and 60°C for 60 sec. Signal was collected at the endpoint of each cycle. The gene expression ΔC_T_ values for each sample were calculated by normalizing with internal control RNU6B, and relative quantitation values were plotted.

### Flow cytometric analysis

Single cell suspensions of metformin and/or FuOx treated or untreated CR HT-29 cells were subjected to direct immunofluorescence staining followed by flow cytometric analysis according to standard protocol [38]. Briefly, the cells were harvested and washed with PBS. Half a million cells were suspended in 90 µl PBS containing 0.5% BSA. After 10 min incubation at room temperature, 10 µl fluorophore (PE-Cy7 or PerCP-Cy5) conjugated ant-human CD44 or CD166 antibody was added and incubated for 30 min in dark at room temperature. The samples were then washed and analyzed using a FACS DiVa (BD, San Jose, CA). The cells stained with IgG2b (isotype-negative control) served as gating control. The proportion of CD44^+^/CD166^+/low^ cells was determined on the basis of fluorescence intensity-spectra.

### Tumor growth in SCID mice

To determine whether the combination of metformin and FuOx would be an effective therapeutic strategy for colon tumors, SCID mice xenograft model of colon tumor was utilized. To generate colon tumors, four weeks old female SCID mice, purchased from Taconic Laboratory, were injected s.c. either with 1×10^6^ CR HCT-116 or CR HT-29 cells suspended in 100 µl Matrigel. They were then divided into 2 subgroups of 4 mice and treatment with metformin was started 7 days after inoculation of the cells in one subgroup. Metformin (RIOMET 500 mg/5 ml; Ranbaxy Laboratories, Princeton, NJ) was dissolved in 100 mL drinking water to attain the dosage of 200 mg/kg body weight. The water was changed daily and measured for water intake. Metformin treatment was continued for 5 weeks until the mice were sacrificed. Metformin group was also injected IP with a mixture of 25 mg/kg 5- fluorouracil and 2 mg/kg oxaliplatin once a week for 3 weeks. Tumors were measured once a week and tumor volumes were calculated using the formula: tumor volume  =  length × width × width/2. Mice were regularly monitored for any signs of discomfort. All animal experiments were performed according to the Wayne State University's Institutional Animal Care and Use Committee (IACUC) approved protocol # A02-02-13. Animal Welfare Assurance # A3310-01.

### Single cell isolation from the xenograft

Small portions (5–10 mg) of the tumor, generated in SCID mice by CR HCT-116 and CR HT-29 cells, as described below were washed extensively in 1× PBS containing 10% antibiotic/antimycotic (AB/AM) and subsequently incubated overnight in Dulbecco's Minimum Essential Media (DMEM/F12) containing 5% antibiotic/antimycotic at 4°C. The tissue was cut into fine pieces using sterile scalpel and then digested with 1.5 mg/ml collagenase I (Sigma-Aldrich) and 20 µg/ml hyaluronidase I (Sigma Aldrich) under gentle agitation for 2–3 hr at 37°C. The digested tissue was filtered through 40 µ filter and centrifuged at 1200 rpm for 5 min. The supernatant (containing dead cells as well as the fat cells) was discarded and the cells (pellet) were washed three times with DMEM/F12 media containing 5% AB/AM. The cells were suspended in previously defined stem cell media.

### Statistical analysis

All *in vitro* experiments were repeated thrice in triplicates. Statistical analysis was performed using Microsoft Excel. The p-values were calculated using 2 sample t-test, assuming unequal variances. Values <0.05 were considered statistically significant.

## Results

### Metformin and FuOx combination therapy inhibits growth of chemo-resistant HT-29 and HCT-116 cells synergistically

**Figure 1:**
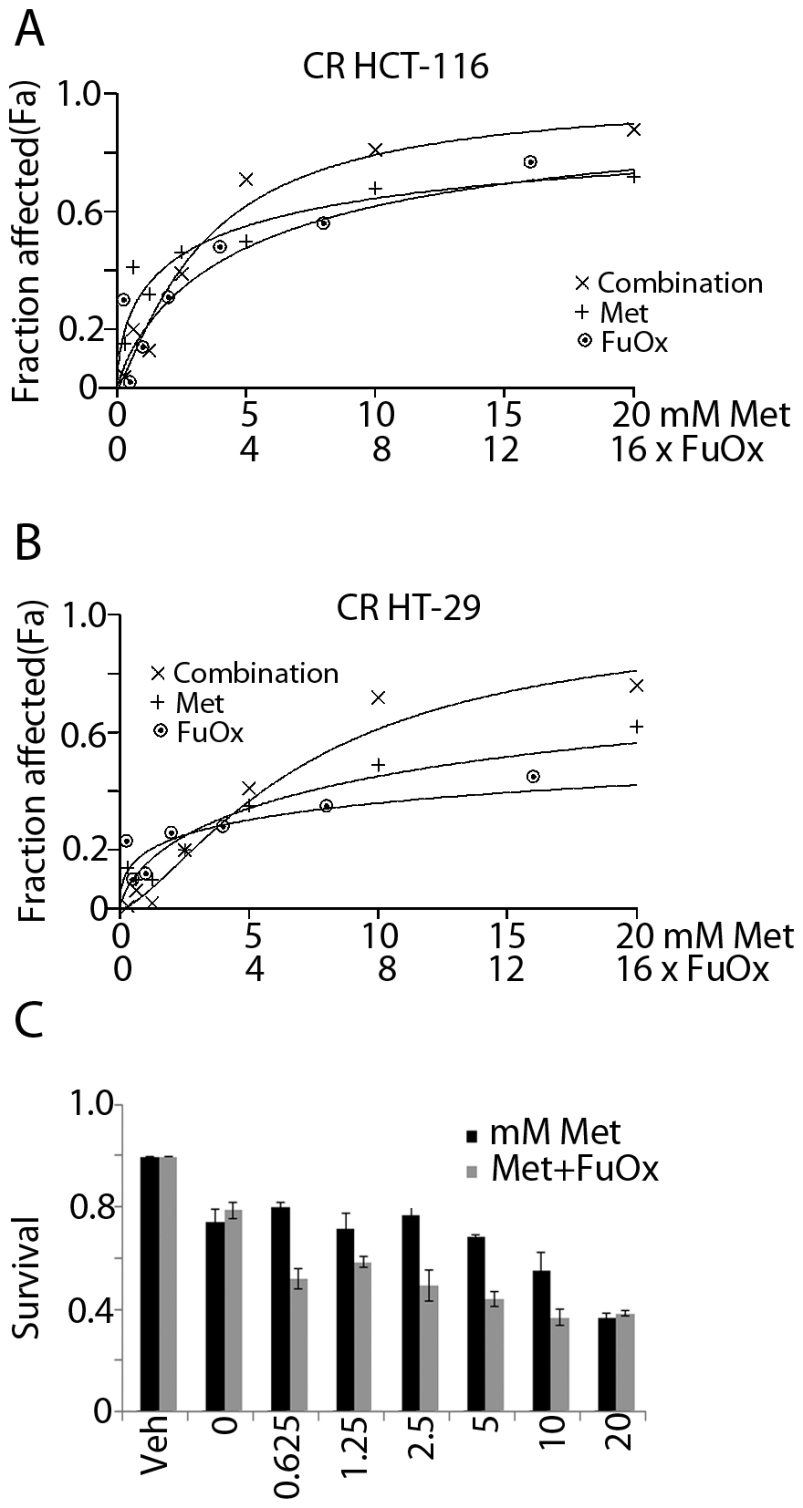
A&B Dose response curves for metformin and/or FuOx in CR HCT-116 (A) and CR HT-29 (B) cells produced by fixed ratio method. Fraction of cells affected by combination of the two drugs (fixed ratio) was higher than either agent alone. Fa represents the fraction of cells affected in response to the treatment. Fa values were used to conduct synergy analysis by CalcuSyn software as described in Materials and Methods. **C**. Relative survival of CR HT-29 cells in the presence of increasing doses of metformin with and without 2×FuOx. Combination treatment is more effective at all the concentrations. Veh: vehicle alone. Each treatment was performed in quadruplicates, bars represent mean ± standard deviation. * p value<0.05.

To examine whether metformin acts synergistically with FuOx to inhibit the growth of chemo-resistant cells, the cells were treated with incremental doses of metformin and FuOx, each alone or in combination. After 72 hr, the cell growth was determined by MTT assay. Dose response curves Fig 1A & B show that while 10 mM metformin or 8× (200 µM FU and 5 µM Oxaliplatin) FuOx alone caused ∼40 and 60% inhibition, together they caused an inhibition of ∼70% and 80% in CR HT-29 and CR HCT-116 cells respectively. The results show that the combination of metformin and FuOx acts synergistically to inhibit the growth of both CR HCT-116 and CR HT-29 cells. All subsequent experiments were carried out using one of the chemo-resistant colon cancer cell lines.

The fractions of cells affected in response to each treatment, was utilized to perform synergy analysis with Calcusyn software. The CI as formulated by the software, revealed values indicating moderate synergistic interaction between the two agents in CR HCT-116 cells at metformin doses 5 mM and higher and a strong synergy at 10 and 20 mM metformin in the CR HT-29 cells (Table 1). No synergism was seen in the parental cells (data not shown). As the chemo-resistant cells are maintained in 2×FuOx (50 µM FU, 1.25 µM oxaliplatin), next we studied the effect of doses of metformin ranging from 0.6 to 20 mM in combination with 2×FuOx on the growth of CR HT-29 cells. The results show that at all the concentrations used, the combination therapy was more effective in inhibiting cell growth than metformin alone (Fig. 1C). The chemo-resistant cells were more resistant to the FuOx and more sensitive to combination therapy compared to the parental cells (data not shown).

**Table 1:** Extreme limiting dilution analysis of colonospheres forming frequency of metformin treated CR HT-29 cells.

Number of Cells/well	Number of wells plated	Number of wells showing colonospheres					
		Control	FuOx	5 mM Met	10 mM Met	5 mM Met ±FuOx	10 mM Met ± FuOx
100	24	24	24	24	24	21	21
10	24	17	13	15	14	5	5
1	24	7	2	0	5	2	0
Sphere forming frequency (95%CI)		1/6(1/10–1/4)	1/12(1/21–1/8)	1/11(1/19–1/7)	1/9(1/15–1/6)	1/43(1/67–1/29)	1/48(1/74–1/31)

### Metformin and FuOx combination therapy inhibits colonosphere formation (number of stem cell/stem like cells)

**Figure 2:**
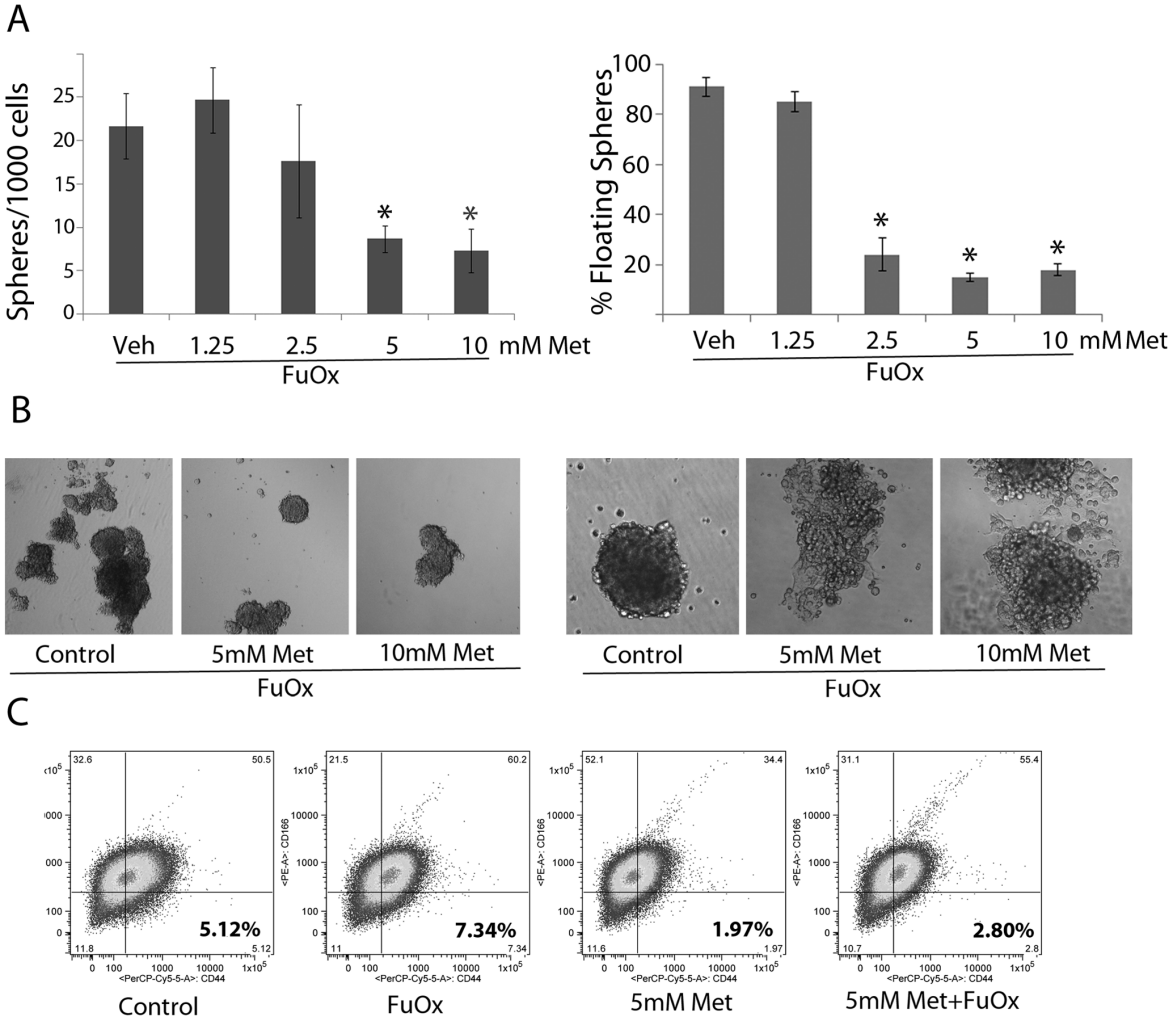
Decreased stem cell characteristics in metformin/FuOx treated CR HT-29 cells. A&B: Formation of primary colonospheres in the presence of metformin and FuOx (left panel). Induction of differentiation and attachment of colonospheres in the presence of metformin/FuOx (right panel). A: graphic representation of the numbers B: photomicrographs of representative spheres. C: Flow cytometric analysis of CD44 and CD166 positive cells after metformin/FuOx treatment.

It was reported that FuOx surviving cells are enriched in CSCs/CSLCs that grow as large, round unattached floating spheroid colonies (colonospheres) when cultured in serum free stem cell medium at a relatively low density [38] [29]. Figs. 2A and B (left panel) show that in the presence of 2×FuOx, increasing concentrations of metformin decreased the number and size of colonospheres. Metformin also induced attachment and disintegrated the colonospheres (Fig. 2A and B (right panel)) When the colonospheres were trypsinized and seeded in the presence of metformin+FuOx, increasing number of spheres lost their stem-like properties as they are seen to attach to the bottom of the dish.

We also evaluated the cancer stem cell properties of the treated cells by performing an extreme limiting dilution assay (ELDA). CR HT-29 cells were treated with metformin+FuOx for 72 hr and seeded in normal stem cell medium. While metformin alone reduced the frequency to form colonospheres by 1.5–2 folds, the combination treatment reduced it by 7–8 folds that of the untreated control (Table 2).

**Table 2:** Combination indices for metformin and FuOx combination therapy, as computed by Calcusyn for CR HCT-116 and CR HT-29 colon cancer cells.

Combination Therapy		Combination Index (CI)	
Metformin mM	FUOX (x)	CR HCT-116	CR HT-29
0.31	0.25	26.818	1544.930
0.62	0.5	2.646	19.756
1.25	1	12.488	961.606
2.5	2	2.351	3.746
5	4	0.602	0.931
10	8	0.537	0.194
20	16	0.499	0.277

To determine whether and to what extent the current treatment strategies would affect the proportion of CSCs/CSLCs, flow cytometric analysis was performed following metformin and/or FuOx treatment of CR HT-29 cells. The results revealed a reduction (∼65%) in CD44^high^/CD166^low^ phenotype cell population, when compared with FuOx treated controls. In contrast, the proportion of CD44^high^/CD166^low^ cells increased from 5.12 to 7.34% in the presence of FuOx, which could, in part, be due to death of FuOx sensitive cells. Combination treatment reduced the proportion of CD44^high^/CD166^low^ cells to 2.8% (Fig 2C). Taken together, these results suggest that the combination of metformin and FuOx not only decreases CR colon CSC/CSLC population, but also diminishes their stem cell properties.

### Metformin and FuOx combination therapy inhibits cell migration

**Figure 3:**
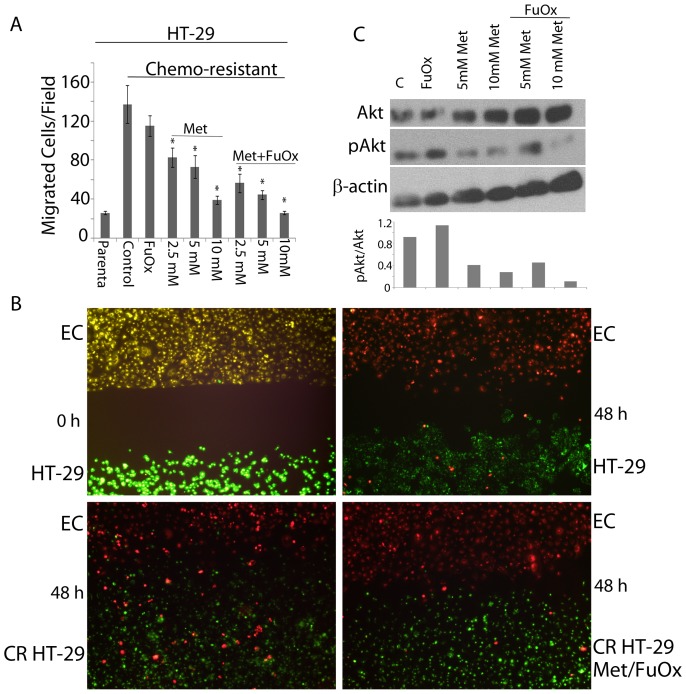
Boyden chamber analysis of migration of parental and CR HT-29 cells. Metformin/FuOx treatment reduced the migration of CR HT-29 cells. Each point represents an average of 6 readings. Bars represent mean ± standard deviation (A). Wound healing assay using endothelial (top/red) and CRC (bottom/green). Top left panel: wound at 0 hr; bottom left panel: complete wound healing at 48 hr in CR HT-29 cells; top right panel: partial wound healing in HT-29 cells after 48 hr; bottom right panel: incomplete wound healing in CR HT-29 cells in the presence of 5 mM metformin/FuOx at 48 hr (B). Western blot analysis of metformin/FuOx treated CR H29 cells indicating decreased pAkt levels (C); relative changes in phospho-Akt (pAkt) were calculated as a ratio of pAkt/Akt after normalization to β-actin, which was used as loading control. *p<0.05.

Cell migration was determined by Boyden chamber (Fig 3A) and wound healing (Fig 3B) assays. Boyden chamber assay indicates that while parental HT-29 cells show limited migration, CR HT-29 cells exhibit strong migration towards Matrigel. However, metformin was found to inhibit migration in a dose dependent manner, and the combination therapy caused further reduction: at 10 mM metformin+FuOx, ∼6 fold inhibition in migration was observed in CR HT-29 cells (Fig 3A).

Wound healing assay was performed to study the migration of parental and CR HT-29 cells towards endothelial cells. Fig. 3B showed CR HT-29 and endothelial cells migrate towards each other. By 48 hr, the wound was completely healed by colon CR cells, while parental cells showed only ∼46% wound closure (Fig.3B). Treatment with metformin+FuOx slowed down the wound healing process. While 54% and complete wound closure was observed in untreated CR HT-29 cells at 24 and 48 hr respectively, metformin+FuOx treatment resulted in a 46 and 77% wound closure at 24 and 48 hr respectively. These results further confirm that metformin+FuOx combination is effective in restraining the tumorigenic/migratory/invasive properties of chemo-resistant colon cancer cells.

It has been reported that Akt is an essential down-stream target of PI3K for the remodeling of actin filaments to increase cell migration [47]. This prompted us to analyze Akt/pAkt expression in metformin and FuOx treated cells. Western blot analysis revealed decreased levels of pAkt in treated cells, compared to controls (Fig. 3). We suggest that PI3K/Akt signaling pathway may play a role in regulating the metformin/FuOx-induced inhibition of cell migration.

### Metformin and FuOx combination therapy decreases miRNA21 and increases miR145 expression

**Figure 4:**
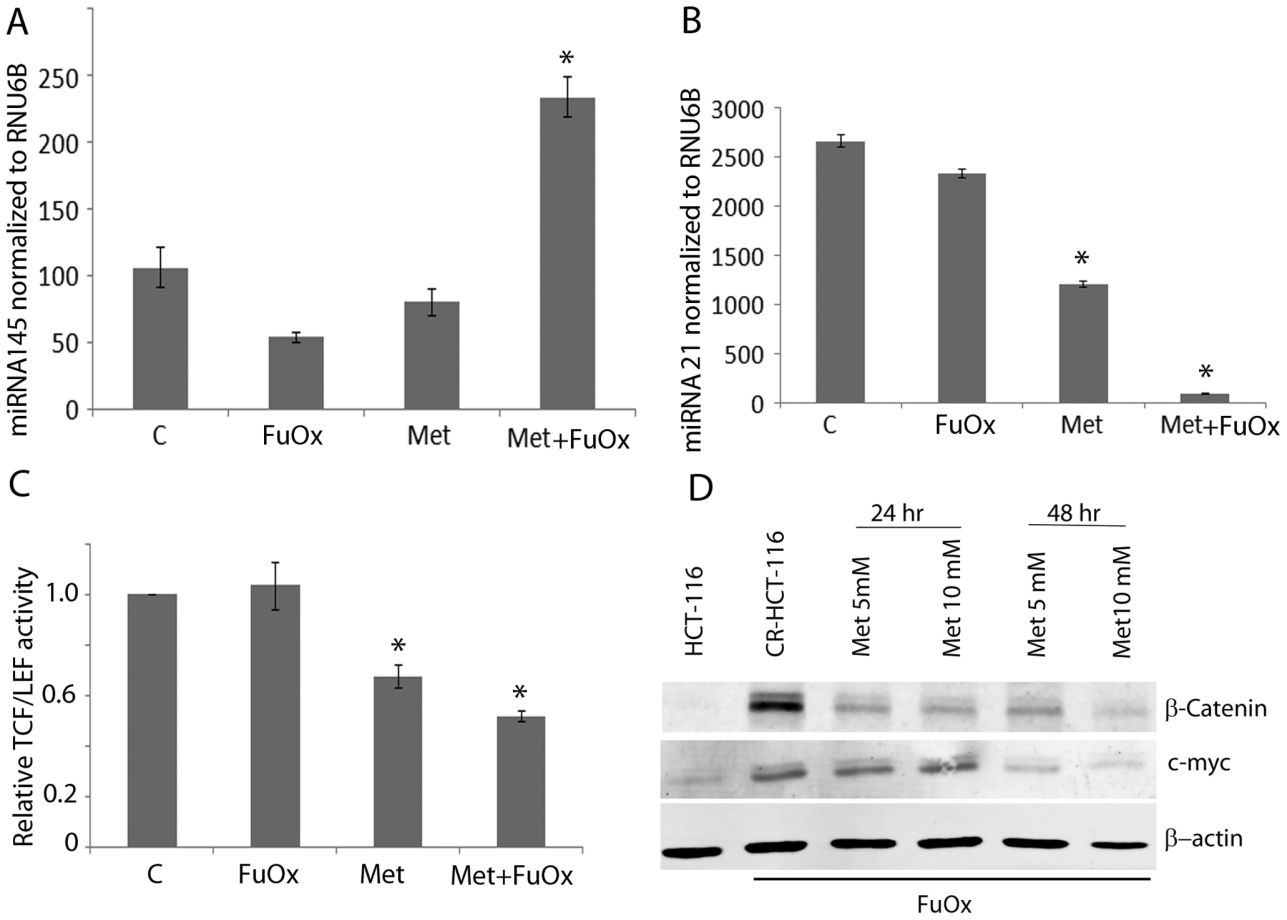
Quantitative real-time PCR showing the expression of miRNA145 (A) and miRNA21(B) normalized to RNU6B in metformin and/or FuOx treated CR HT-29 cells. C: metformin/FuOx treatment of CR HCT-116 cells inhibits transcriptional activity of TCF/LEF. D: Western blot analysis shows a reduced expression of β-catenin and c-myc in metformin/FuOx treated CR HCT-116 cells. β-actin was used as loading control. * p<0.05.

In view of the notion that miR-21 is oncogenic, while miR-145 is tumor suppressive, we have analyzed their levels in chemo-resistant colon cancer cells. We found the levels of miR21 to be higher in CR colon cancer cells [33] and miR145 to be lower, when compared with the corresponding parental controls (unpublished data). Results of the quantitative real time PCR analysis showed, while metformin either alone or together with FuOx caused a marked reduction of miR-21expression, it induced miR-145, when compared to their respective controls (Fig. 4). Suffice it to mention that the qRT-PCR values for miR-21 and miR-145 were normalized to the levels of RNU6B.

As the Wnt/β-catenin signaling plays a critical role in the regulation of colon cancer stem cell proliferation, we next studied the effect of metformin treatment on β-catenin activity, its expression and the levels of its target protein c-myc. The combination treatment of metformin and FuOX caused ∼50% decrease in the transcriptional activity of TCF/LEF in CR HCT-116 cells, compared to the untreated controls (Fig 4C). Western blot analysis showed a marked decrease in the levels of total β-catenin as well as c-myc expression in CR HCT-116 cells. Taken together, the results suggest an inhibition of Wnt/β-catenin signaling in chemo-resistant colon cancer cells in response to the combination therapy of metformin and FuOx.

### Metformin and FuOx combination therapy retards tumor growth in SCID mice

**Figure 5:**
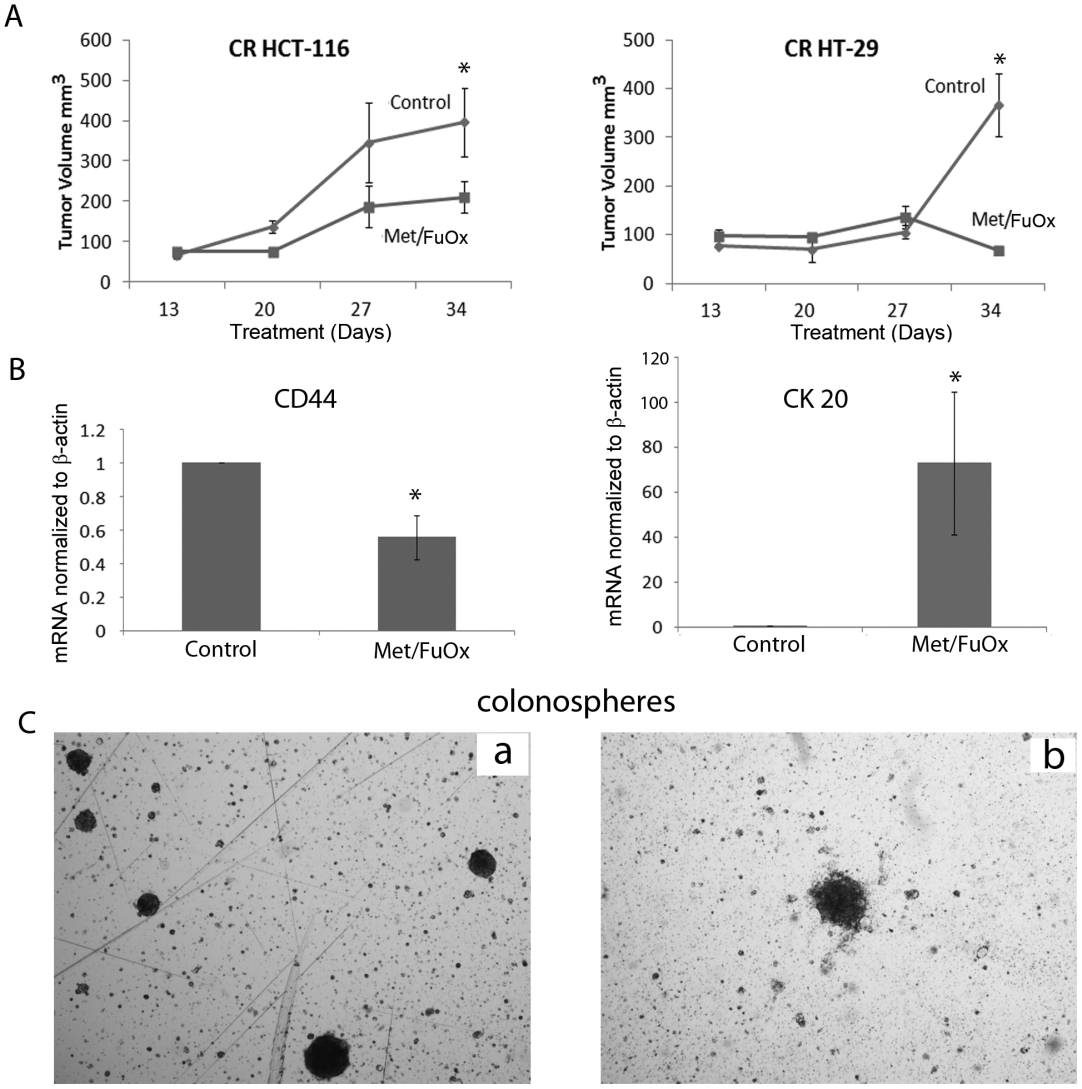
Tumor growth in metformin/FuOx treated SCID mice injected with CR HCT-116 (left pane) or CR HT-29 cells (right panel) (A). Bars represent mean ± standard error. * p >0.05. Real time quantitative PCR on RNA extracted from cells isolated from the CR HT-29 tumor (B). CD44 levels decreased and CK20 levels increased in metformin/FuOx treated xenografts. Colonosphere formation in the cells isolated from CR-HT-29 xenografts (C).

To examine the therapeutic effectiveness of the combination of metform and FuOx, the SCID mice xenograft model of colon tumor was utilized. CR HCT-116 or CR HT-29 cells were injected subcutaneously in the flank region of SCID mice. After palpable tumors were formed, one group was fed metformin in the drinking water and was also injected (i.p) with FuOx, once a week for 3 weeks. Tumor growth was measured by calculating tumor volume for 34 days. Xenografts formed by CR HCT-116 cells showed a linear growth till 27 days, after which the tumor growth slowed down. A similar growth pattern was seen in metformin/FuOx treated mice, albeit the growth was significantly lower compared to controls. By day 34 post-injection, there was a statistically significant (almost 50%) reduction in tumor size in metformin/FuOx treated mice (Fig 5A left panel). CR HT-29 cells injected mice showed a slow tumor growth rate initially. However, a growth spurt was seen in control animals after 27 days. On the other hand, metformin/FuOx treated mice showed a rapid decline in tumor volume after 27 days.

The tumors were harvested, and single cell suspensions were cultured in stem cell medium. Real-time qPCR analysis was performed, which showed a significant decrease in CD44 and increase in CK20 mRNA levels in tumor cells form metformin/FuOx-treated mice (Fig 5B). Metformin/FuOx-treated xenografts also had a reduced number of stem/stem like cells as evidenced by the colonosphere formation (Fig 5C).

## Discussion

The current investigation was performed in an attempt to develop new therapeutic strategies for recurrent/metastatic CRC, where the effectiveness of metformin in combination with the conventional chemotherapy was analyzed. We found that metformin acts synergistically with FuOx to inhibit the growth of chemo-resistant HCT-116 and HT-29 cells.

Earlier *in vitro* studies have demonstrated an inhibition of proliferation of colon cancer cells by metformin [48]. *In vivo* studies have demonstrated that metformin delays tumor onset in mouse model for *p53* mutant colon cancer [49] and inhibits colon carcinoma growth stimulated by a high-energy diet [50]. Metformin was also shown to significantly suppress colonic epithelial proliferation by inhibiting the mammalian target of rapamycin (mTOR) pathway [18], [51]. In a randomized clinical trial among non-diabetic patients, metformin treatment decreased the number of aberrant crypt foci significantly compared to the un-treated group with a follow-up of 1 month [52]. Another study performed on diabetic patients with colorectal cancer showed decreased cancer-related mortality in patients using metformin [53]. In breast cancer xenografts Hirsch *et al* [19] showed that metformin treatment specifically eliminated CD44^+^/CD24^−/low^ stem cells synergistically with doxorubicin. Similarly, in prostate and lung adenocarcinomas, metformin inhibited growth of cancer stem cells [54]. None of these studies however, focused on the recurrent colorectal cancer, which is resistant to the conventional chemotherapy.

Although the reasons for CRC recurrence are not fully understood, it is thought to be related to inability of the conventional chemotherapy to target CSCs/CSLCs [9]. Recent characterization of CSC/CSLCs has led to the identification of key cellular events that among others include epithelial to mesenchymal transition (EMT), drug efflux capability, anti-apoptotic mechanisms and induction of differentiation [55]. Stem cell signaling pathways could, therefore, be targeted to increase the vulnerability of CSCs/CSLCs to therapeutic regimen. Indeed, data from our *in vitro* cell culture studies show an increased cell death in CR colon cancer cells in response to the combination of metformin and FuOx. A similar growth inhibitory effect of metformin/FuOx treatment was also observed *in vivo*, as evidenced by the reduction in tumor volume of xenografts of CR HCT-116 and CR HT-29 cells in SCID mice. Clearly, these results suggest a therapeutic effectiveness of metformin and FuOx combination in tumors caused by chemo-resistant colon cancer cells.

Earlier, we reported a predominance of CSCs/CSLCs in CR colon cancer cells that show increased ability to exclude drugs, and elevated expression of CSC markers such as CD44, CD166, CD133 and ALDH [38], [43], [56]. Our observation that inhibition of tumor growth in SCID mice by the combination of metformin and FuOx is associated with reduction in CD44 levels and colonosphere forming ability in cells derived from xenograft of CR cells indicates reduction in CSCs/CSLCs. Although the mechanisms for reduction in CSCs/CSLCs are not fully known, our observation of a marked increase in CK-20 expression in colon tumors of metformin/FuOx-treated SCID mice suggests differentiation of CSCs/CSLCs.

Further support for the observation that metformin/FuOx treatment effectively targets colon CSCs/CSLCs and eventually recurrent CRC, comes from the *in vitro* data, which demonstrates that this therapeutic combination inhibits not only the expression of colon CSC markers, but also colonosphere formation and induces their disintegration. Drug resistance and the emergence of CSCs in tumor cells have been attributed in part to the phenomenon of EMT [57]–[61], that confer on it survival and migratory advantages [57], [62] resulting in invasive and metastatic properties (reviewed in [10]). Inhibition of migration in CR cells further indicates that the current combinatorial treatment reduces not only the growth, but also cell migration associated with EMT and invasive properties of CR CSCs/CSLCs. The fact that combination of metformin and FuOx markedly inhibits TCF/LEF activity and decreases β-catenin and c-myc levels suggests that Wnt/β-catenin signaling, which is greatly activated in many malignancies, including CRC [23], plays a pivotal role in regulating the tumor growth.

Recent evidence suggests a role for miRNAs in the regulation of proliferation, apoptosis, differentiation, metabolism and invasion by simultaneously regulating the post-transcriptional expression of numerous genes [63], thereby also playing an important role in CSC function, which involves a complex interplay between multiple pathways. Chemo-resistance in HCT-116 and HT-29 cells was reported to be associated with increased expression of the miR21, an oncomiR [38], [43] and reduced expression of the tumor suppressor miR145 (unpublished data). Recently metformin was reported to enhance the cytotoxic effects of 5-FU in HCT-116 cells via regulating miR21 [64]. Our observation that metformin and FuOx treatment reduced the levels of miR21 and increased miR145 in CR HT-29 cells indicates the reversal of chemo-resistant/CSC/CSLC characteristics in colon cancer by modulating miR-21 and miR-145.

Metformin is emerging as a multi-faceted drug. Suppression of mTOR through AMPK activation is believed to constitute the major mechanism underlying its anti-cancer activities [12], [17], [48], [65]–[68]. The results from the current investigation indicate that metformin acts synergistically with FuOx and inhibits cell proliferation, migration and tumor growth of chemo-resistant colorectal cancer cells partly via affecting the viability of CSCs/CSLCs by modulatig miRNA 21, miRNA 145, and Wnt/β-catenin signaling. In summary, to the best of our knowledge this is the first investigation that shows the therapeutic effectiveness of metformin and FuOx combination therapy on recurring colorectal cancer and demonstrates that the current treatment strategy could be effective against chemo-resistant colon cancer cells that are highly enriched in CSCs/CSLCs.
